# IL-38 Gene Deletion Worsens Murine Colitis

**DOI:** 10.3389/fimmu.2022.840719

**Published:** 2022-05-26

**Authors:** Dennis M. de Graaf, Ruth X. Wang, Jesús Amo-Aparicio, J. Scott Lee, Alexander S. Dowdell, Isak W. Tengesdal, Carlo Marchetti, Sean P. Colgan, Leo A. B. Joosten, Charles A. Dinarello

**Affiliations:** ^1^Department of Medicine, University of Colorado Denver, Aurora, CO, United States; ^2^Department of Internal Medicine, Radboud University Medical Center, Nijmegen, Netherlands; ^3^Mucosal Inflammation Program, Department of Medicine, University of Colorado School of Medicine, Aurora, CO, United States; ^4^Medical Scientist Training Program, University of Colorado School of Medicine, Aurora, CO, United States; ^5^Department of Medical Genetics, Iuliu Hatieganu University of Medicine and Pharmacy, Cluj-Napoca, Romania

**Keywords:** IL-38, NLRP3, IL-1α, IL-1β, colitis, IBD – inflammatory bowel diseases

## Abstract

IL-38 is a recently discovered cytokine and member of the IL-1 Family. In the IL-1 Family, IL-38 is unique because the cytokine is primarily a B lymphocyte product and functions to suppress inflammation. Studies in humans with inflammatory bowel disease (IBD) suggest that IL-38 may be protective for ulcerative colitis or Crohn’s disease, and that IL-38 acts to maintain homeostasis in the intestinal tract. Here we investigated the role of endogenous IL-38 in experimental colitis in mice deficient in IL-38 by deletion of exons 1-4 in C57 BL/6 mice. Compared to WT mice, IL-38 deficient mice subjected to dextran sulfate sodium (DSS) showed greater severity of disease, more weight loss, increased intestinal permeability, and a worse histological phenotype including increased neutrophil influx in the colon. Mice lacking IL-38 exhibited elevated colonic *Nlrp3* mRNA and protein levels, increased caspase-1 activation, and the concomitant increased processing of IL-1β precursor into active IL-1β. Expression of IL-1α, an exacerbator of IBD, was also upregulated. Colonic myleloperoxidase protein and *Il17a*, and *Il17f* mRNA levels were higher in the IL-38 deficient mice. Daily treatment of IL-38 deficient mice with an NLRP3 inhibitor attenuated diarrhea and weight loss during the recovery phase. These data implicate endogenous IL-38 as an anti-inflammatory cytokine that reduces DSS colitis severity. We propose that a relative deficiency of IL-38 contributes to IBD by disinhibition of the NLRP3 inflammasome.

## Introduction

Ulcerative colitis (UC) and Crohn’s disease (CD) are characterized by chronic hyperinflammation of the gastrointestinal tract (1). An innate inflammatory response is elicited in healthy subjects upon recognition of pathogen-associated molecular patterns (PAMPs) and danger-associated molecular patterns (DAMPs) *via* pattern recognition receptors (2). In inflammatory bowel disease (IBD) patients with susceptible genetics a disproportional, auto-inflammatory response is triggered by environmental factors. As a result, the mucosal barrier is impaired and leads to symptoms of abdominal pain, fatigue, weight loss, diarrhea, and intestinal bleeding (3, 4). Currently, IBD treatments range from glucocorticoids to thiopurines, antibodies targeting cytokines and integrins, small-molecule drugs, and fecal- and stem-cell transplants (5). However, these therapies fail to induce a response in a portion of patients and lose efficacy in those who initially benefit.

The nucleotide-binding oligomerization domain-like receptor family, pyrin domain-containing 3 (NLRP3) forms an inflammasome with the adaptor molecule apoptosis-associated speck-like (ASC) protein containing a caspase recruitment domain and pro-caspase-1 (6). The NLRP3 inflammasome drives the innate immune response against pathogenic infections, by cleaving precursor IL-1β and IL-18 precursors into active cytokines (7). NLRP3 in the gut promotes intestinal health by contributing to barrier integrity and pathogen clearance (8). However, in several inflammatory diseases, NLRP3 activity is dysregulated and contributes to disease severity (9). In IBD patients, for example, disease severity is associated with upregulation of IL-1β, NLRP3, and caspase-1 (10). Indeed, murine colitis induced by dextran sodium sulfate (DSS) is exacerbated by *Nrlp3* deficiency when compared to WT mice (11, 12).

IL-38 is a recently discovered anti-inflammatory IL-1 Family member that is abundantly expressed in keratinocytes of the skin and (circulating) B lymphocytes (13–15). In humans, the gene encoding IL-38 (*IL1F10*) is located within the IL-1 gene cluster on chromosome 2p13, adjacent to the genes encoding receptor antagonists IL-1Ra and IL-36Ra with which IL-38 shares 41% and 43% homology, respectively. Similar to IL-36Ra, IL-38 inhibits IL-36 signaling by inhibiting the IL-1R6, which reduces the Th17 response (16). Notably, IL-17 expression in the mucosa and serum of IBD patients is upregulated (17).

IL-38 expression in colon samples from patients with active UC and CD is increased compared to age-matched healthy controls (18). The IL-38 expression is confined to infiltrating immune cells in the lamina propria, mucosa, submucosa, muscular and serosa layers, epithelial and parenchymal cells (18), and CD123+ cells (19). Furthermore, IL-38 staining in the lamina propria was detected CD19+ B cells but absent in CD3+ T cells or CD68+ monocytes and CD14+ macrophages (18, 20). In a separate study, IL-38 expression is highest in colonic tissue from non-inflamed patients with UC, i.e., those in remission, compared to patients with active UC, CD and healthy subjects (19). This finding is in stark contrast to the expression of other members of the IL-36 subfamily including IL-36Ra, which was upregulated in inflamed colonic tissue from patients with active UC (19). Recently Xie et al. demonstrated that recombinant IL-38 is protective in a murine model of dextran sulfate sodium (DSS) colitis (18). Thus, endogenous IL-38 may have a role in the maintenance of gut homeostasis and tissue repair. We recently reported that recombinant IL-38 reduces *Nlrp3* gene expression and promotor accessibility in mouse bone marrow (21) and can limit IL-1β production in the synovium of mice subjected to gouty arthritis (22). As of this writing, no relationship between IL-38 signaling and the NLRP3 inflammasome activity has been described in IBD.

Here, we studied the role of endogenous IL-38 in a murine model of DSS colitis by comparing responses in WT to those in IL-38 deficient mice, focusing particularly on the involvement of the NLRP3 signaling. We hypothesize that endogenous IL-38 contributes to IBD resolution and intestinal homeostasis by limiting NLRP3 expression and activity.

## Materials and Methods

### Ethics Statement

Animal protocols were reviewed and approved by the University of Colorado Animal Care and Use Committee.

### Mice

**Figure 1:**
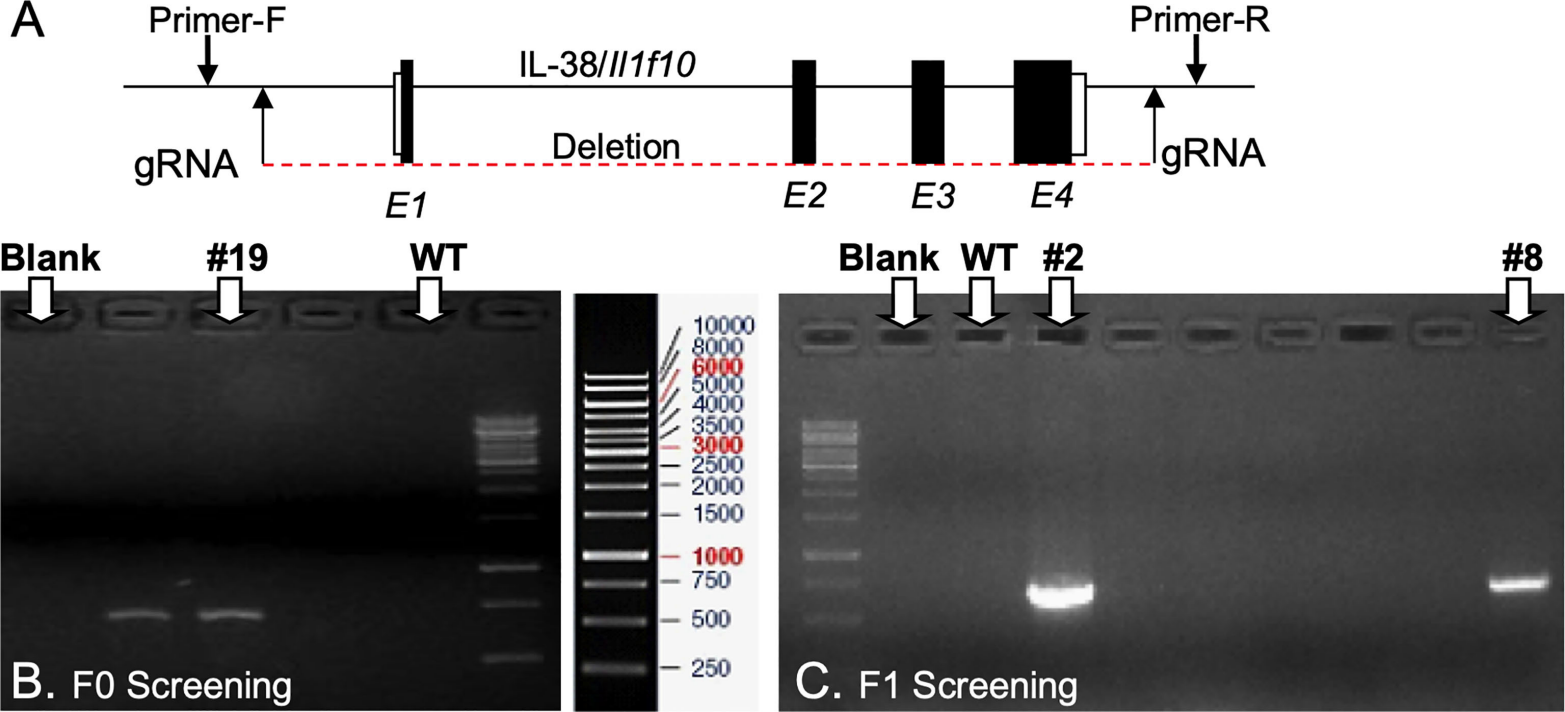
Generation of IL-38/*Il1f10* deficient mice. **(A)** Representation of the IL-38 gene, exons are in black, gRNA and PCR primer locations are indicated with arrows. F0 **(B)** and F1 **(C)** Heterozygote *Il1f10* deficient founder screening. PCR products were generated using primers in **Table 1** and conditions listed in the **Supplemental Material**.

*Il1f10* (IL-38) deficient mice (GenBank accession number: NM_153077.2; Ensembl: ENSMUSG00000046845) were generated using CRISPR/Cas9 technology (Cyagen Biosciences, CA, USA) on a C57BL/6 background. **Table 1** contains gRNA sequences used for the removal of *Il1f10* exons 1-4 and nucleotides to confirm the deletion. *Cas9* mRNA and gRNA were generated by *in vitro* transcription and injected into fertilized C57BL/6 eggs. Founders were genotyped by PCR using TaKaRa TaqTM Hot Start Version (Takara) and the PCR product was purified using the MiniBEST Universal Genomic DNA Extraction Kit Ver.5.0, 9765 (Takara). The IL-38 deficiency was confirmed by PCR and gel electrophoresis, and DNA sequencing analysis (**Figure 1**). DNA sequencing revealed that F0 Mouse-ID #19, and F1 Mouse-ID #2 and #8 were missing 4725 bases in one *Il1f10* allele, indicating loss of all *Il1f10* exons. After transportation to our animal facility and further breeding, presence of homozygote IL-38 deficient knockout or wild type (WT) alleles were confirmed by ear clippings through Transnetyx prior to 3 weeks of age. All mice used in this study were between 8 and 10 weeks of age. All national and institutional guidelines for the care and use of laboratory animals were adhered to.

**Table 1:** gRNA and PCR primer sequences.

Purpose	Sequence	Vector ID
gRNA before *Il1f10* exon 1 (matches fwd. strand)	GCGAGAGAACAGTTACCGAATGG	VB180116-1110yca
gRNA after *Il1f10* exon 4 (matches fwd. strand)	TTCAATATTGGTAGGCACCCCGG	VB180116-1112eat
Identification of IL-38 deficiency (Short product indicates deletion of exons 1-4)	Fwd: CCCATGCCGTAGAGCACATCTGT Rev: GGCTCATCTTGTGCTGTAGCTCTGC	
PCR Identification of WT (Transnetyx)	Fwd: TCCAGGGTACCTGAGCTTCA Rev: GTCTTCTCATGAGGTAATTGAGGATGT	
PCR Identification of knockout (Transnetyx) (presence indicates *Il1f10* deficiency)	Fwd: CTGGCTCATTGCTTGTAACACTAC Rev: AGGAGAATCCCTAGATGTCTTTCCA	

### Dextran Sodium Sulfate Colitis

Male and female mice were subjected to DSS at 8-10 weeks of age. From day 0, mice received drinking water with 3% DSS (molecular weight 36,000–50,000; MP Biomedicals) to induce acute colitis, or H_2_O as a vehicle control. After 5 days of treatment, mice were allowed to recover for 4 days on regular drinking water before sacrifice. A disease activity index (DAI) score was assessed daily to evaluate the development of colitis based on the parameters of weight loss compared to initial weight, stool consistency, and rectal bleeding (23). Evaluation was performed by two researchers who were blinded to the experimental groups. Scores were defined as weight loss: 0 (0%), 1 (1-5%), 2 (5-10%), 3 (11-20%), and 4 (>20%); stool consistency: 0 (well-formed pellets), 2 (pasty, semi-formed pellets), and 4 (liquid stools); and rectal bleeding: 0 (no blood), 2 (hemoccult positive), and 4 (gross bleeding). The highest DAI score possible was 12 (24). Colon lengths were measured at time of sacrifice, and tissue collected for histology, immunofluorescence, RNA, and protein analyses. For select experiments, mice lacking IL-38 and exposed to DSS for 5 days were treated daily i.p. with 200 mg/kg OLT1177, a specific NLRP3 inhibitor, or saline as a control, for the duration of the experiment, based on a previous study (25).

### Histological Scoring

Distal colon tissue was fixed in methacarn (methanol:chloroform:acetic acid, 60:30:10) and stained with hematoxylin and eosin. All histological scoring was performed blinded. Three independent parameters were assessed: severity of inflammation (0-3: none, slight, moderate, severe), depth of injury (0-3: none, mucosal, mucosal, and submucosal, transmural), and amount of crypt damage (0-4: none, basal 1/3 damaged, basal 2/3 damaged, only surface epithelium intact, entire crypt and epithelium lost). The independent parameter scores were multiplied by a factor reflecting the percentage of tissue involved (x1: 0-25%, x2: 26-50%, x3: 51-75%, x4: 76- 100%) and then added up. Maximum histological score possible was 40 (26).

### Colon Permeability

Six hours before the sacrifice, mice received 100 μL of 100 mg/mL 70-kDa FITC-dextran (Sigma-Aldrich) by oral gavage, which only transits a permeable gut barrier. Blood was collected 4h later, centrifuged at 1000 x *g* for 10 min, and serum was analyzed for FITC fluorescence and compared to a FITC-dextran standard.

### RT-qPCR

TRIzol reagent (ThermoFisher Scientific) was used to isolate total RNA from colon tissue. cDNA was prepared using the iScript cDNA Synthesis Kit (Bio-Rad). Quantitative PCR analysis was performed using the Power SYBR Green master mix (Applied Biosystems) in a thermocycler. Fold change in expression of target mRNA relative to *Gapdh* mRNA was calculated using the delta-delta Ct method. *Gapdh* F: 5′-TTCAACAGCAACTCCCACTCTTCCA-3′, *Gapdh* R: 5′- ACCCTGTTGCTGTAGCCGTATTCA-3′ *Il17a* F: 5′- TTTAACTCCCTTGGCGCAAAA-3′, *Il17a* R, 5′-CTTTCCCTCCGCATTGACAC-3′ *Il17f* F: 5′- TGCTACTGTTGATGTTGGGAC-3′, *Il17f* R, 5′- AATGCCCTGGTTTTGGTTGAA-3′, *Nlrp3* F: 5′- TGGTATGCCAGGAGGACAGCCT 3′, *Nlrp3* R: 5′- AGACGCGCGTTCCTGTCCTT -3′.

### ELISA

Colon samples were rinsed with PBS and lysed in 200 μL of radioimmunoprecipitation assay (RIPA, 50 mM Tris-HCl pH 8.0, 1 mM EDTA, 1% Triton X-100, 10% SDS, 0.5% sodium deoxycholate, 150 mM NaCl, Sigma-Aldrich) buffer with protease inhibitors on ice. Samples were homogenized by sonication, and insoluble materials removed by centrifugation at 10,000 x *g* for 5 min at 4°C. Total protein was quantified using the Bradford Assay (Bio-Rad). ELISA on colon protein extracts was performed for IL-1α, IL-1β and myeloperoxidase (MPO), and on plasma for KC/CXCL1 according to the manufacturer’s instructions (Biotechne).

### Western Blot

Colon protein extractss were electrophoresed on Mini-PROTEAN TGX 4−20% gels (Bio-Rad) and transferred to nitrocellulose 0.2 μM (GE Water & Process Technologies). Membranes were blocked in 5% dried milk in 0.5% PBS-T for 1 hour at room temperature. Primary antibodies for caspase-1 1:500 (sc-514 Santa Cruz Biotechnology, Dallas, TX, USA), and Nlrp3 1:1000 (Adipogen, San Diego CA) were used in combination with peroxidase-conjugated secondary antibodies and chemiluminescence to detect the protein. A primary antibody against β-actin (Santa Cruz Biotechnology) was used to assess protein loading. Bands were quantified using ImageJ (Maryland, USA).

### Statistical Analysis

The data represents the mean ± SEM, #P < 0.1, *P < 0.05, **P < 0.01, ***P < 0.001, ****P < 0.0001 by two-way ANOVA with Fisher’s multiple comparison, or Student’s T-test.

## Results

### Loss of IL-38 Exacerbates the Phenotype of DSS Colitis

**Figure 2:**
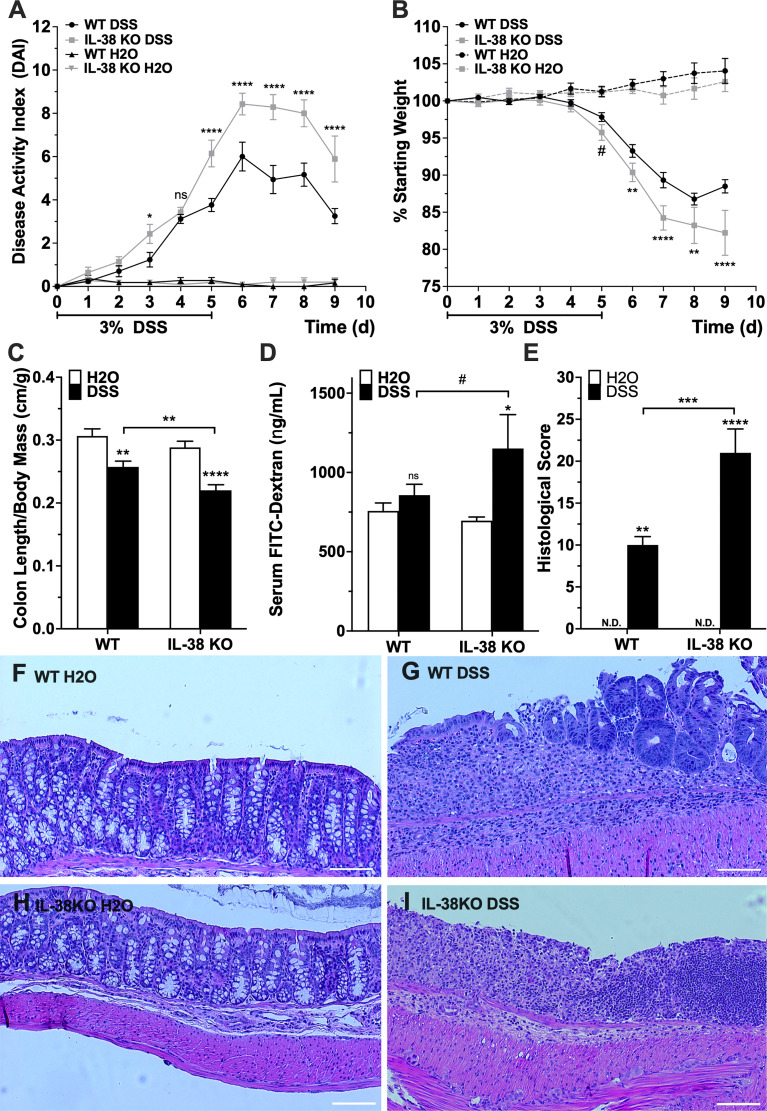
IL-38 deficiency aggravates DSS colitis. **(A)** DAI scores of WT and IL-38 deficient mice [indicated as knock-out (KO) mice]. Mice were subjected to DSS or H_2_O only. Maximum DAI is 121. N = 11 for H_2_O, 14 for DSS. **(B)** Weight loss of WT and IL-38 KO mice subjected to DSS versus H_2_O only. Maximum loss is 20%. N = 11 for H_2_O, N = 14 for DSS. **(C)** Colon length to body mass ratios of WT and IL-38 KO mice subjected to DSS versus H_2_O only. N = 8-9. **(D)** Serum FITC-dextran in WT and IL-38 KO mice subjected to DSS versus H_2_O (n = 6-7). **(E)** Histological scoring of colon tissue of WT and IL-38 KO mice subjected to DSS versus H_2_O. Maximum histological score 40 (n = 6). **(F-I)** Representative H&E stained histological slide of colon tissue of WT mice subjected to H2O **(F)** or DSS **(G)**, and IL-38 deficient mice subjected to H2O **(H)** and DSS **(I)**. Data were pooled from 2-3 separate experiments. The data represents the mean ± SEM, ^#^P < 0.1, *P < 0.05, **P < 0.01, ***P < 0.001, ****P < 0.0001 by two-way ANOVA with Fisher’s multiple comparison. N.d., Not detectable; ns, not significant.

To investigate the influence of endogenous IL-38 to outcomes of experimental colitis, WT and IL-38 deficient mice were subjected to 3% DSS or water control for 5 days, allowed to recover for 4 days and sacrificed on day 9. As shown in **Figure 2A**, IL-38 deficient mice displayed enhanced susceptibility to DSS colitis compared to WT mice, as demonstrated by significantly higher DAI scores at day 3 (P < 0.05) and days 5-9 (P < 0.0001). The DAI in WT and IL-38 deficient plateaued from days 6 to 8 and recovery began on day 8. Moreover, mice lacking IL-38 had a greater loss of bodyweight than WT mice from day 6 onwards (P < 0.01) (**Figure 2B**). Upon sacrifice, IL-38 deficient mice revealed a greater reduction in colon length/weight ratio compared to WT control (P < 0.01, **Figure 2C**), whereas this ratio was not dissimilar between vehicle-treated WT and IL-38 deficient mice (P = 0.27).

To assess intestinal barrier permeability, mice received 70 kDa FITC-dextran by oral gavage on the last day of the experiment, and FITC-dextran was measured in serum after 4h. DSS treated IL-38 deficient mice had an enhanced permeability defect compared to vehicle (H_2_O) treated IL-38 deficient mice (P < 0.05), and IL-38 deficient colitic mice trended towards having an enhanced barrier defect compared to WT colitic mice (P < 0.1) (**Figure 2D**).

Colons were assessed histologically after sacrifice. As presented in **Figure 2E**, colitic IL-38 deficient mice had increased histologic scores that reflected the severity of inflammation, depth of injury, and crypt damage compared to WT mice (P < 0.001). Representative images in **Figures 2F–I** demonstrate a complete loss of crypt morphology associated with inflammatory leukocyte infiltration and severe epithelial damage in mice lacking IL-38 mice. In contrast, DSS treated WT mice displayed a modest level of inflammation and associated epithelial and crypt damage. Overall, a deficiency in IL-38 amplified the disruption of the intestinal barrier function and intensified colitic disease. No histological differences between WT and IL-38 deficient mice were observed at baseline.

### IL-38 Deficiency Enhances Inflammatory Cytokine Production in DSS Colitis

**Figure 3:**
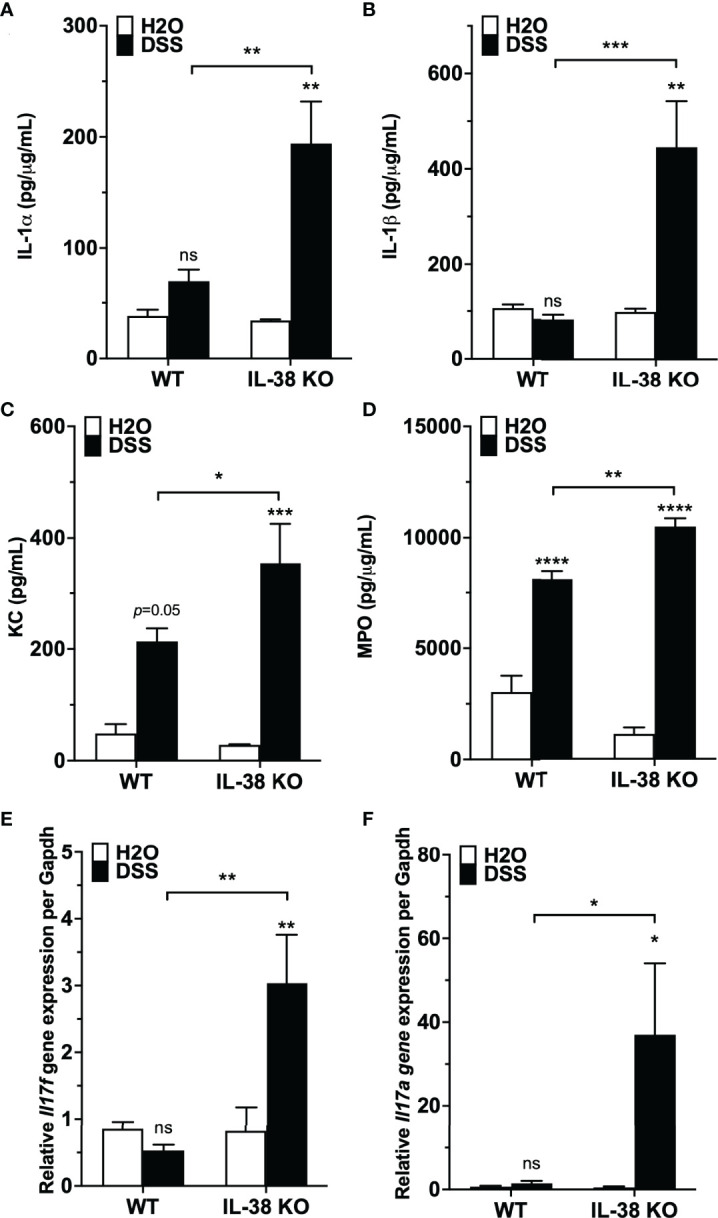
Mice lacking IL-38 have increased expression of inflammatory cytokines in colitis. WT and IL-38 KO mice were subjected to H_2_O or DSS. **(A, B)** IL-1α, IL-1β protein expression in colon tissue, **(C)** plasma KC concentration, **(D)** MPO protein expression in colon tissue, **(E)** mRNA levels of *Il17a* and **(F)** *Il17f* in colonic tissue measured by qPCR, normalized to GAPDH. Data was pooled from 2-3 separate experiments. The data represents the mean ± SEM, *P < 0.05, **P < 0.01, ***P < 0.001, ****P < 0.0001 by two-way ANOVA with Fisher’s multiple comparison. IL-38 KO: IL-38 deficient. ns, not significant.

We investigated the influence of endogenous IL-38 on inflammatory cytokines during colitis. As presented in **Figures 3A, B**, pro-inflammatory IL-1α and IL-1β protein levels were increased in the IL-38 deficient mice compared to WT mice treated with DSS. Plasma concentrations of the neutrophil chemokine KC and colonic expression of the neutrophil activation marker MPO were upregulated in WT mice exposed to DSS in comparison to water controls, and further increased in DSS treated IL-38 deficient mice (**Figures 3C, D**). As shown in **Figures 3E, F**, gene expression of Th17 cytokines *Ill17a* and *Il17f* were upregulated only in IL-38 deficient mice.

### Endogenous IL-38 Reduces Inflammatory Signaling

**Figure 4:**
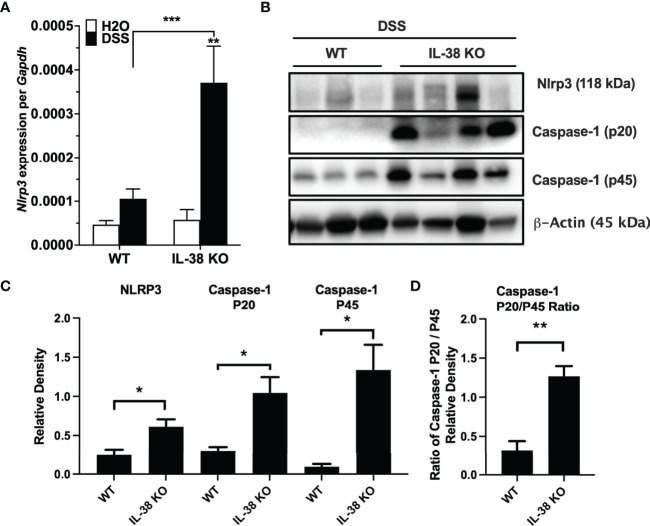
Endogenous IL-38 inhibits NLRP3 activation in colitis. WT and IL-38 KO (IL-38 deficient) mice subjected to DSS. **(A)** mRNA expression of *Nlrp3* in colonic tissue measured by qPCR, normalized to GAPDH. **(B)** NLRP3, Caspase-1 (p20), Caspase-1 (p45) and β-Actin protein abundance in colonic tissues quantified by Western Blot analysis. Lanes 1-3 contain colonic tissue of WT mice treated with DSS, lanes 4-7 from IL-38 deficient mice subjected to DSS. A representative blot is shown from two experiments with 3-4 mice per group. **(C)** Quantifications of blots presented in **(B)**. **(D)** P45/p20 ratio in WT mice treated with DSS compared to IL-38 KO mice. The data represents the mean ± SEM, *P < 0.05, **P < 0.01, ***P < 0.001, by two-way ANOVA with Fisher’s multiple comparison in **(A)**, and student’s T-test in **(C, D)**.

**Figure 5:**
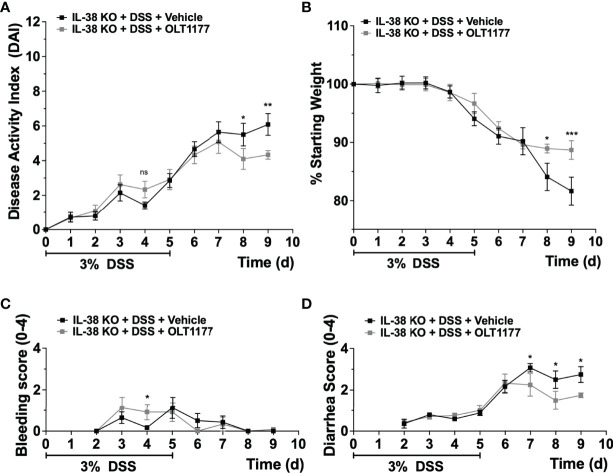
NLRP3 inhibition enhances recovery in IL-38 deficient mice. IL-38 deficient mice were subjected to DSS and treated daily i.p. with 200 mg/kg OLT1177, an NLRP3 inhibitor, or vehicle (N = 7 per group). **(A)** DAI scores, maximum DAI is 12. **(B)** Weight loss, maximum loss is 20%. **(C)** Bleeding score, maximum score is 4. **(D)** Diarrhea score, maximum score is 4. Data were pooled from 2 separate experiments with 3-4 mice each. The data represents the mean ± SEM, *P < 0.05, **P < 0.01, ***P < 0.001, by two-way ANOVA with Fisher’s multiple comparison. Ns: not significant.

Next, we investigated the effect of endogenous IL-38 on the expression of NLRP3 and caspase-1 in mice subjected to DSS. As shown in **Figure 4A**, *Nlrp3* gene expression was significantly increased in IL-38 deficient mice. Western blot analysis revealed an increase in protein expression Nlrp3, pro-caspase 1 (p45), cleaved caspase-1 (p20) (**Figures 4B, C**) and the ratio of cleaved-to precursor caspase-1 (**Figure 4D**) in the colon of IL-38 deficient mice subjected to DSS in comparison to WT mice. These findings indicate that endogenous IL-38 is associated with inhibition of NLRP3 inflammasome. In H2O-treated WT and IL-38 deficient mice, Nlrp3 protein expression was nearly undetectable, whereas IL-38 deficient mice had increased baseline caspase-1 cleavage (**Supplementary Figure 1**). To investigate whether the exacerbated colitis phenotype in the IL-38 deficient mouse depended on NLRP3 activity, mice were treated with vehicle or an NLRP3 inhibitor for the duration of DSS treatment and recovery. As presented in **Figures 5A–D**, NLRP3 inhibition in IL-38 deficient mice leads to more DSS-induced bleeding and disease activity on day 4. However, less disease activity was observed during the last days of the recovery phase due to a reduction in diarrhea and weight loss. These data indicate that NLRP3 activation during the recovery phase delays the recovery from DSS-induced colitis in IL-38 deficient mice.

## Discussion

In the present paper, we examined whether IL-38 deficiency amplifies the colitis response using the DSS model. The data indicate that the IL-38 deficient mouse has an increased disease activity score, weight loss, histological damage, and intestinal permeability. The aggravated colitis in mice lacking IL-38 was accompanied by a greater increase in the pro-inflammatory cytokines IL-1α and IL-1β in the colon compared to WT mice. These observations are supported by a previous publication showing that treatment with recombinant IL-38 reduces DSS induced colitis (18).

We observed that the IL-38 deficient mouse exhibited consistently increased expression of the neutrophil chemokine KC in the plasma and the neutrophil activation marker MPO in the colon. We also observed an increase in colonic *Il17a* and *Il17f* mRNA expression (**Figure 3**). Relevant to these data, IL-1R1 deficient mice were reported to have a reduced colonic Th17 response and IL-17 secretion (10), which is in line with endogenous IL-38 reducing colonic IL-1β and IL-1α protein expression, thereby reducing IL-1R1 signaling. Neutrophil influx, here a factor contributing to the disease activity index, was also elevated in IL-38 deficient mice, as indicated by an increase in MPO expression and increased leukocyte influx as part of the histological scoring. This finding corroborates previous observations of recombinant IL-38 reducing leukocyte influx into the synovium of mice subjected to gouty arthritis (22) and neutrophils into lesional skin in a mouse model of psoriasis (14, 27).

The measurement of inflammatory cytokines was performed on samples obtained at the time of sacrifice, i.e., 4 days after the mice were withdrawn from DSS. At that time point, the WT mice subjected to DSS had an increase in KC and MPO in comparison to WT mice that received vehicle treatment with H_2_O, but were without an increase in IL-1α and IL-1β protein, and *Il17a*, and *Il17b* gene expression. To unequivocally determine the effect of endogenous IL-38 on either the induction or recovery phase of DSS-induced colitis, inducible knock-out/knock-in IL-38 mice may be generated in which IL-38 deficiency occurs only during each specific phase.

The IL-38 deficiency that aggravated DSS colitis was associated with elevated gene expression and protein levels of NLRP3 inflammasome and enhanced IL-1β signaling at the time of sacrifice. The IL-38 deficient mouse also had increased expression of pro- and active caspase-1, a prerequisite for IL-1β processing. At baseline, mice lacking IL-38 had increased colonic caspase-1 activation. In IL-38 deficient mice treated with DSS, inhibition of NLRP3 resulted in more bleeding on day 4, but overall less disease activity due to reduced weight loss and diarrhea in the last days of the recovery phase. Thus, the amplified disease phenotype observed during the recovery phase in the IL-38 deficient mice could be partially rescued by NLRP3 inhibition. Previous reports on blockade of NLRP3 with this specific NLRP3 inhibitor effectively limited the induction of DSS colitis in mice (28), and inhibits NF-κB, IL-1β, caspase-1 and MPO activity (25). In line with our observations, NLRP3 deficient mice treated with DSS were reported to have increased rectal bleeding on days 3 and 4 (29). Notably, a detrimental role of NLRP3 in DSS colitis has also been reported (11), indicating that the contribution of NLRP3 to DSS-induced colitis may be different between the induction- and recovery phase of this model.

Under homeostatic conditions, the NLRP3 inflammasome aids epithelial barrier integrity and has antimicrobial activity (8). In disease, excessive NLRP3 and the IL-1β precursor expression are induced by NF-κB through DAMPs and PAMPs (30). PAMPs associated with the gut microbiota make their way through the epithelial barrier and bind pattern recognition receptor expressing cells. DAMPs such as IL-1α are released from epithelial cells undergoing cell death (31). Relevantly, Bersudsky et al. reported that IL-1β from myeloid cells promotes healing and repair in DSS-induced colitis, unlike IL-1α which is primarily inflammatory (32). In IBD, colonic IL-1β expression correlates positively with disease activity, particularly in active lesions (33). Genetic polymorphisms in *NLRP3* are associated with increased risk of CD (34) and UC (35, 36). The inhibition of NLRP3 by IL-38 has recently been demonstrated in an *in vitro* model of temporomandibular joint inflammation (37).

Several studies report on the role of IL-36 family members in mouse models of IBD. In mice subjected to DSS colitis, IL-36α/γ and IL-38 gene expression are increased in the colon during the peak of colitis, whereas IL-36β, IL-36Ra, and IL-1R6 remained stable (38, 39). Yang et al., recently reported that deficiency of the IL-1R6 agonist IL-36γ leads to hypo-responsiveness to DSS-induced colitis, whereas the IL-36Ra deficient mouse is hyper-responsive (40). These data are in line with our observations of amplified colitis in the IL-38 deficient mouse, and are consistent with IL-38 and IL-36Ra both inhibiting IL-1R6 signaling. Interestingly, IL-1R6 deficient mice display decreased DSS-induced colitis compared to WT mice through reduced infiltration of neutrophils and macrophages (38), yet also have a defective recovery (41). The detrimental effects of endogenous IL-1R6 signaling during the induction of colitis by DSS, and its beneficial role during the recovery phase, may be dependent on the balance of pro- and anti-inflammatory IL-36 cytokines during each phase, which requires further investigation.

In IBD, patients with no detectable serum IL-38 most often had measurable CRP concentrations, although this negative correlation was not significant (18). Circulating IL-38 levels of overweight individuals with chronic low-grade inflammation are inversely correlated to CRP, TNF, IL-6 and leptin (42). Relevantly, CRP itself can induce NLRP3 activation (43), and conversely IL-38 may reduce the induction of CRP by inhibiting NLRP3.

The gut microbiome also plays a key role in IBD (44). Blockade of IL-1α with a neutralizing antibody protects mice from acute DSS-induced colitis and modifies the gut microbiota into an anti-inflammatory flora (31). Here, we show that endogenous IL-38 reduces IL-1α expression in mice subjected to DSS. Hence, a thorough characterization of the microbiota of IL-38 deficient mice, or perhaps human subjects with a relative IL-38 deficiency, will help determine whether loss of endogenous IL-38 results in gut microbiota alterations that exacerbates the inflammatory phenotype reported in these animals. Our data indicate that mice lacking IL-38 that received normal drinking water did not show a difference from vehicle treated WT mice, indicating that there is no spontaneous inflammatory phenotype.

The expression of IL-38 in IBD patient samples is abundant throughout the mucosa, submucosa, muscular, and serosa layers (19). IL-38 gene expression is significantly higher in active UC compared to active CD (19). The analysis of colonic biopsy specimens showed that levels of IL-36α/γ and IL-38, but not IL-36β, are increased in active CD patients and related to IL-1β and IL-17A (39). IL-36 precursor levels are enhanced in active lesions in UC patients (19, 33). IL-36Ra expression in lamina propria mononuclear cells is significantly reduced in the colon of UC patients (38), and DNA microarray analysis has identified IL-36γ as the most preferentially expressed cytokine in inflammatory colonic macrophages (45).

IBD patients show increased risk for developing colorectal cancer (CRC), due to the pro-carcinogenic effects of chronic inflammation (46, 47). Wang et al. observed that IL-38 gene expression was reduced in CRC tissues in comparison to healthy tissue, and high IL-38 expression in CRC biopsies was associated with prolonged survival and smaller tumor size (48), indicating that therapeutic use of recombinant IL-38 in CRC deserves investigation. Furthermore, the IL-38 associated SNP rs6734328 was also related to a reduced risk for CRC (49). This implies that chronic reduced expression, or deficient endogenous IL-38 likely contributes to both IBD and CRC development and progression.

## Concluding remarks

We demonstrate that endogenous IL-38 reduces the inflammatory phenotype associated with IBD in a mouse model of DSS colitis. Our data suggest that during the recovery from colitis, IL-38 deficient animals have detrimental NLRP3 activity, and NLRP3 inhibition attenuates the recovery process. We propose that a relative deficiency of IL-38 contributes to IBD by disinhibition of the NLRP3 inflammasome.

## Data Availability Statement

The original contributions presented in the study are included in the article/**Supplementary Material**. Further inquiries can be directed to the corresponding author.

## Ethics Statement

The animal study was reviewed and approved by University of Colorado Animal Care and Use Committee.

## Author Contributions

DG, RW, SC, and CD designed research; DG, RW, JA-A, JL, AD, IT, and CM performed research; DG and RW analyzed data; DG drafted the manuscript paper. RW, JA-A, SC, LJ and CD corrected the manuscript. All authors contributed to the article and approved of the submitted version.

## Funding

DG and CM are supported by the Interleukin Foundation. RW is supported by an NIH National Research Service Award (NRSA) fellowship F30DK120072 and NIH Medical Scientist Training Program (MSTP) training grant T32GM008497. JL is supported by NIH grant DK129410. CD is supported by NIH Grant AI-15614. SC is supported by NIH grants DK1047893, DK50189, and DK095491.

## Conflict of Interest

LJ serves on Olatec’s scientific advisory board and receives compensation. CD serves as chairman of Olatec’s scientific advisory board, is co-chief scientific officer, receives compensation, and has equity in Olatec. CM serves as director for Olatec’s innovative science program and has equity in Olatec.

The remaining authors declare that the research was conducted in the absence of any commercial or financial relationships that could be construed as a potential conflict of interest.

## Publisher’s Note

All claims expressed in this article are solely those of the authors and do not necessarily represent those of their affiliated organizations, or those of the publisher, the editors and the reviewers. Any product that may be evaluated in this article, or claim that may be made by its manufacturer, is not guaranteed or endorsed by the publisher.

## Supplementary Material

Supplementary Figure 1 Endogenous IL-38 expression is associated with reduced colonic caspase-1 activation. WT and IL-38 KO (IL-38 deficient) mice subjected to H2O. NLRP3, Caspase-1 (p20), Caspase-1 (p45) and β-Actin protein abundance in colonic tissues quantified by Western Blot analysis. Lanes 1-3 contain colonic tissue of WT mice treated with H2O, lanes 4-6 from IL-38 deficient mice subjected to H2O. A representative blot is shown from two experiments with 3 mice per group.

## Abbreviations

ASC, apoptosis-associated speck-like; CD, Crohn’s Disease; DAI, Disease Activity Index; DAMP, Danger Associated Molecular Pattern; DSS, Dextran Sulfate Sodium; IBD, Inflammatory Bowel Disease; NLRP3, nucleotide-binding oligomerization domain-like receptor family; pyrin domain-containing 3; PAMP, Pathogen Associated Molecular Pattern; UC, Ulcerative Colitis; WT, wild type.
