# Supplementary figures and images for: IL-38 Gene Deletion Worsens Murine Colitis
Source: Front Immunol. 2022 May 26;13:840719. doi: 10.3389/fimmu.2022.840719 (PMC9181991; doi:10.3389/fimmu.2022.840719)

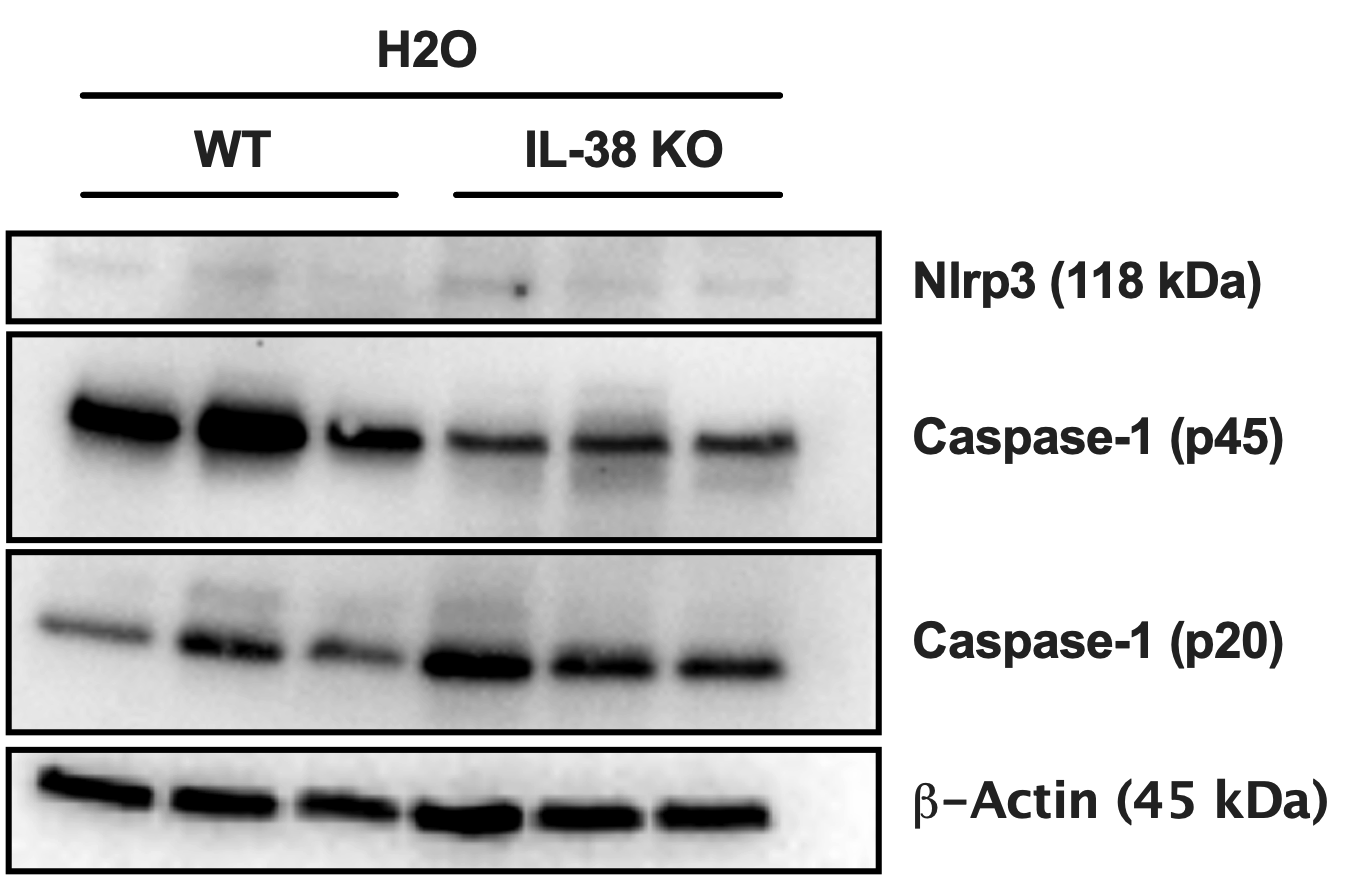

Supplement: Supplementary Figure 1 — Endogenous IL-38 expression is associated with reduced colonic caspase-1 activation. WT and IL-38 KO (IL-38 deficient) mice subjected to H2O. NLRP3, Caspase-1 (p20), Caspase-1 (p45) and β-Actin protein abundance in colonic tissues quantified by Western Blot analysis. Lanes 1-3 contain colonic tissue of WT mice treated with H2O, lanes 4-6 from IL-38 deficient mice subjected to H2O. A representative blot is shown from two experiments with 3 mice per group. [file Image_1.tiff]
