# Supplementary material for: IL-38 Gene Deletion Worsens Murine Colitis
Source: Front Immunol. 2022 May 26;13:840719. doi: 10.3389/fimmu.2022.840719 (PMC9181991; doi:10.3389/fimmu.2022.840719)
Supplement: Supplementary file 2 [file DataSheet_1.docx]

***Supplementary Material***

**Founder screening genotyping**

PCR primers: Mouse Il1f10-F: 5’-CCCATGCCGTAGAGCACATCTGT-3’, Il1f10-R: 5’-GGCTCATCTTGTGCTGTAGCTCTGC-3’. Initial denaturation 94 ℃ for 5 min, followed by 35 cycles of denaturation at 94 ℃ for 30s, annealing 30s at 60 ℃, Extension for 60s at 72 ℃. Final extension at 72 ℃ for 5 min and kept at 12 ℃ until gel electrophoresis. Expected Product Size: WT: 5420 bp; KO: ~690 bp, delete~4730 bp. DNA Sequencing Primer (Reverse Sequencing): 5’-GCAGCTCACCCCTCACTAAATCAG-3’. The amplicons were then purified and sent for DNA sequencing analysis.

**Sequencing Result**

Described is the deletion sequence in *Il1f10 (IL‑38)* of 4725 bases of F0-mouse-ID#19 & F1-mice-ID#2, #8. The exons are highlighted in bold, the start codon and stop codon are colored in red.

taccgaatggttcagaagtggggccatgcctgtgactttaaacaaatgtttcatatttttaaataataacacttagattacaaaataaatttactacaggaaaatgttaagaactatcaacaaccattgactatcctgtcggccacaaatgagtgttataacaagcaccagccgtccttgtccacatgtgtgtgtgtctacacagctatgaatttaattgggataataatgtgcacattctttacggcctgcagtttttacttcatgtatttgaaatgtttgtgccacaaatgtcatctttaaggagcatatccttatttcctggatttatcattccctttcagccgactggacattgacagcatttccaacttttcaaccttgtaaaaataactaattgaactattttataactaagcatttgggcaatcaattacctctgcctggaatgggggcaacaacacatgcaatcatgggaaagccaggatgctgctgtctgatccctagccctggcattcgtgcagaacctcactctcatctgtgccctgatatccttcactctcaagtcttttcccagtgacttttaaaggcaacagaatcatatagccaataatgaaagctacttggtctacagttgtgtggcgttttttatagatattttcttcatttacatttcaaatgctatcccaaaagtcccctataccctcccccaccctgctcccctacccactcactcccacttcttggccctggctttcccccttactggggcatataaagtttgctagaccaaggggcctctcttcccaatgatggccaactaggccattttctgctacatatgcagctagagacaccagttctggggttactggttagttcatattgttgttctacctatggggttgcagaccccttcagctcttgagtactttctctagctcctccattgggagccctgtgttccatcctatagatgactgtgagcatccacttctgtatttgccaggcactggcatatgaaatagtatctgcatttggtggctgattatgggatggacccccgggtggggcagtctctggatggtccatcctttcatcttagctccaaactttgtctctgcaacttcttccatggatattttagtccctaatctagggagaaatgaagtatccacaagttgatcttccttcttgattttcttatgttttagaagttgtatcttggatattctaggtttctgggctaatatccacttatcagtgagtacatatcaagtgaattcttttgtgattaggttacctcactcaagatgatattctccactatgttcatagcagccctatttatagtagccagaagctggaaagaacccagtccctcaacagaggaatggatacagaaaatgtggcacatttatgcaatggagtaccactcagatattaaaaacaacgaatttatgaaattctcgggcaaaaccctatctaaagaccaggaataaggaaaagatggactgcctgcctgcagctgggagagctggggagacctttgtggattctgtaatacttaggggtacggaacagcttgtggctggataattctgagctccagcatgtctgccccccaaaaaacattctgtttttctgaaagcctttttcttctttgcctcag**tgaagaccagacactcccaactgcaggaatgtgctcccttcccatggcaagatactacat**gtaagtaatcttaacgatcgctcaatcaaggggcctggagatcacatgagaagggaaaaggctgagtcaaagggacaaagctccctctagccacagaaatctcaaacactgaataattgatcttcatctttgtcaatcacaacagccctctttcctggtgacagaatggaacaactgtaagagtggtattgcttagtccattttacagacccggaaactcaacctccacgaggttatacaattttcctcatgtcatgcaattacccaaaagcagagagtgggatcggactctctgttctctaaactgatgtagctagttcttagaaagctcaaacaatcttgagtcccaaggacagcacctttatggtcacctggattgatacctatatcaaaaaaaaaaaaaggtctcactagatagccctggctaccctgaaactctcactgtgtacatttaggtgaccacgaactcacagagatctgccttccaagtgctgggattaaagtatgtaccaccacacctgcatctttgacaataactgagtggtatctaaattcttccagtggctaaacagttaagtcccagttcccaaagtctgagaaaaatgccaggtggtgaaatctgtacagacctttgttcttaatgtacaagtgagcctgctttaaaaacaatacgcaagctgtttttgctattgctaagtgttgcagagacagaaaaggctcccagaagtggtaactttggtccagaggttctgttctcaaactcattgtgagctctgaaagcaactgatgggcagctctgaaatcagctgggcaattaggctaataacaggcataattttaatgtttcacacgcatgacagttcctccccagctgccctagtacatacttaccctcctaggcacgtcattagacccataggtataaccagtgactaatcaggccctggtctaattctaagttggcctcctatataagtgccactcagagtgtacctcatcatggctgtagtgggcccagagtctagggacatagacttttctattgtccaatttctgatttgtgaattttctacaaaaagaattttttttaattttacaaatcaaatcacagttactacatcttcagttccttcattaattagtgttactatttaaaaaaataaaataaatcaagctcagaaacatcatggatagggttcattgtatctccagggtacctgagcttcaaagcaactcctcagacagccatgaaaacatcctcaattacctcatgagaagacactattgtcatttctggagcctctgataatcctgagcctaggcagctttgggatgaaacaatttctacccttattggaacagtgtccctctcctgtctggaaacaattcaccaaaggctccatgtggttgtccagtaaggtggtatggggacagaaatggacaatgatccctgagggcagtgatccattaaccttgccctcctatttcag**aatcaaggatgcacatcaaaaggctttgtacacacggaatggccagctcctgctgggagaccctgattcagacaattatagtccag**gtgatcttccggtggtgggggtgggggagtggaggggagggtgtggggggggctctcttccagaagttgcttagtgtccatctgccacaaggccttgattctttccttcaattgtgtctctagagacatgagaatattgtcacagtgataaggagaagaggtaggggcagtttcttcctgtaaaaaatgaattccatttaccctgcagtctccatacagaaacaggccagaggggggcagacccagtaacttctagctgagccctaccttgcttaaaacctgccatctgtggtcccctcactgtctgaattgcattctgtcttacctcccag**agaaggtctgtatccttcctaaccgaggcctagaccgctccaaggtccccatcttcctggggatgcagggaggaagttgctgcctggcgtgtgtaaagacaagagagggacctctcctgcagctggag**gtgagacacccctcctcattgcagtcagtactgccactggaacatagtgacatctttgaacccacatgtcccctctcttgtttcccatctatctctctttgcctccagctgagggactctagcctttggggatgtacagaaagaacatggcttcggaaaactcttccctattgagtccttctttggccaagcctctgaggcactaagggctgacgtcccaaccaaacactcatttcatctcacagctgtctccctttccccacag**gatgtgaacatcgaggacctatacaagggaggtgaacaaaccacccgtttcacctttttccagagaagcttgggatctgccttcaggcttgaggctgctgcctgccctggctggtttctctgtggcccagctgagccccagcagccagtgcagctcaccaaagagagtgaaccctccacccatactgaattctactttgagatgagtcggtaaagagacataaggctggggcctcgtctagtgcccccagtctga**gatcttcttgctcagcatctctggaaagcagaataaggaagataccaaagatgtttgggtcttaatccccagaatctgtgaccgtgttacattaaatggcaaagggattttttttttccttcatggtccatttgggcccattggaatcatctgaggcctcatgaggagaaggaagaggtcagagggagactggggcaaactttggtactaaaagtaacaatggagacagggaccataagctgatgggtaacagtggtttctagaaaccggaaatgatgagagctctcctgacacaggttctggatttttctggactgaagaatggtgaaataatacagctccattattttaagccactgagtttgagatcattcaatgaagctgtcataataaaacctgtgcttcacatacaattcaatattggtaggcaccccggt
